# Rapid Treatment of Rhinophyma with Powered Microdebrider

**DOI:** 10.1155/2013/621639

**Published:** 2013-02-21

**Authors:** C. Faris, J. G. Manjaly, H. Ismail-Koch, S. Caldera

**Affiliations:** Department of Otolaryngology and Head and Neck Surgery, Queen Alexandra Hospital, Southwick Hill Road, Cosham, Portsmouth PO6 3LY, UK

## Abstract

We describe here our experience in using sinus microdebrider to rapidly debulk and sculpt the tissues in cases of rhinophyma correction. We utilized the use of the 4 mm M4 Rotatable Cutting Straight Sinus Blade on a straight Straightshot M4 Microdebrider by Medtronic at 800 rpm oscillation which is normally utilised in our sinus surgery practice. The microdebrider is straightforward to use and is already stocked in most ENT departments. It requires no additional training or cost outlay for departments that perform endoscopic sinus surgery with microdebrider. In our experience it affords the surgeon the ability to rapidly and accurately sculpt the nose to an excellent aesthetic result. We feel it is a more precise tool than cold steel or Bovie cautery, quicker than CO_2_ laser techniques, and avoids the aerosol of dermabrasion. No complications occurred in our series, and all patients rated their cosmetic outcome as good to excellent.

## 1. Introduction

Rosacea is a common condition with classic symptoms such as intermittent patchy flushing, erythema, and inflammation. It has a predilection for the face particularly on the cheeks, nose, forehead, and around the mouth. Rosacea typically appears between the ages of 30 and 50 and affects more women than men. It runs a chronic course with a small minority of patient progressing to phymas (Greek = swelling, mass). Rhinophyma occurs almost exclusively in men. In one study of 108 patients with rosacea only 14% of patients were suffering from rhinophyma and 93% were men [1]. The tumour-like phymas progressively enlarge in size causing increasingly overt deformity. Rhinophyma may lead to social stigmatisation as there is a frequent incorrect causal association with the condition being self-inflicted by excess alcohol intake, the “Whiskey nose”, or through facial deformity in severe cases. Over time as the rhinophyma enlarges the excess weight of the phymatous tissue on the alar can lead to collapse of the internal and external nasal valves with resulting functional nasal obstruction. Surgical reduction is indicated when there is significant enlargement of the soft tissues [2]. We describe our experience using rapid sculpting of the phymatous tissues of the nose using the Straightshot M4 Microdebrider normally utilized in sinus surgery in 3 patients.

## 2. Technique

**Figure 1:**
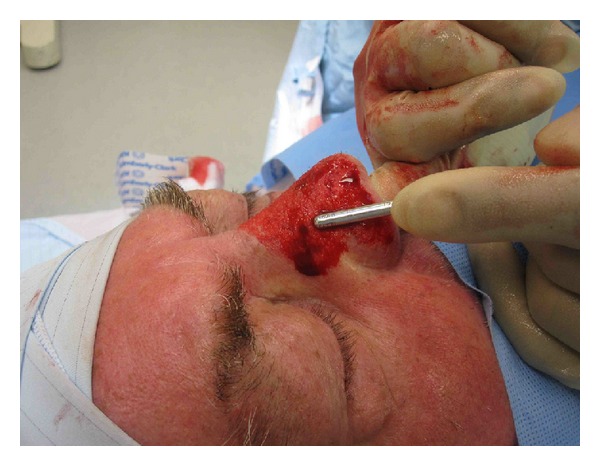
Case 3, technique with bimanual approach to enable maximal tactile feedback, note the microdebrider is held like a pen.

**Figure 2:**
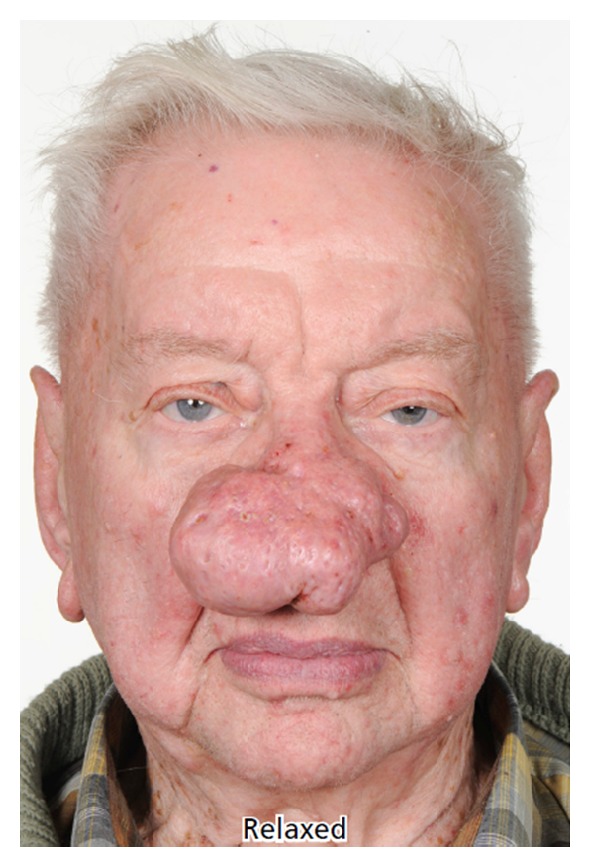
Case 3, preoperative frontal photo.

**Figure 3:**
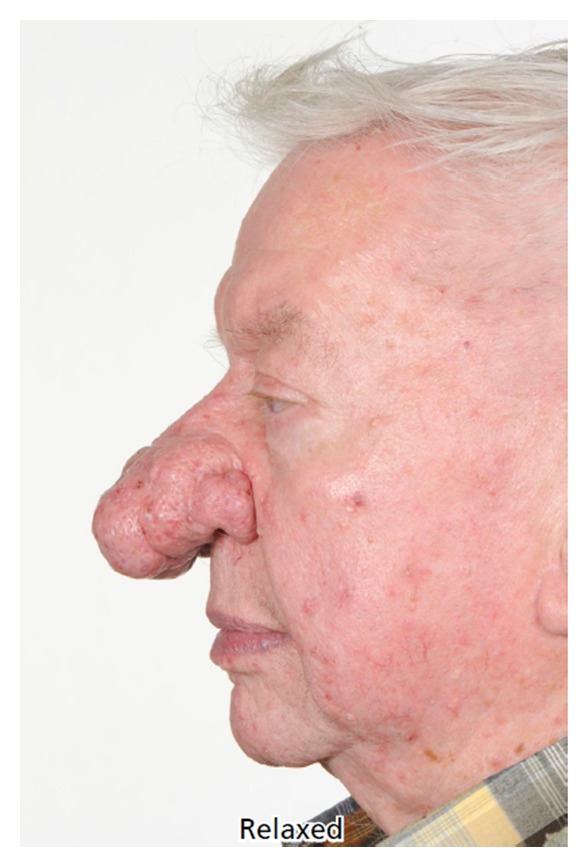
Case 3, preoperative lateral photos.

**Figure 4:**
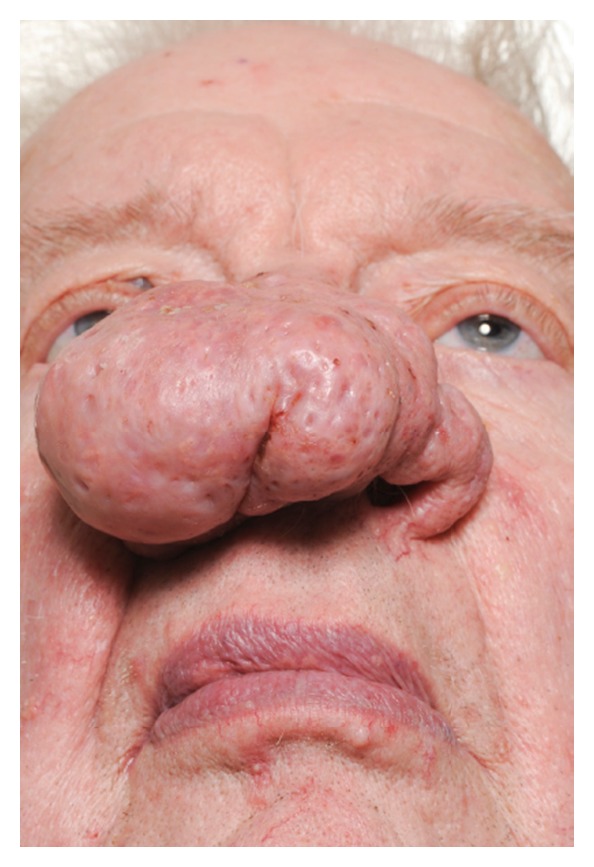
Case 3, preoperative basal photos.

**Figure 5:**
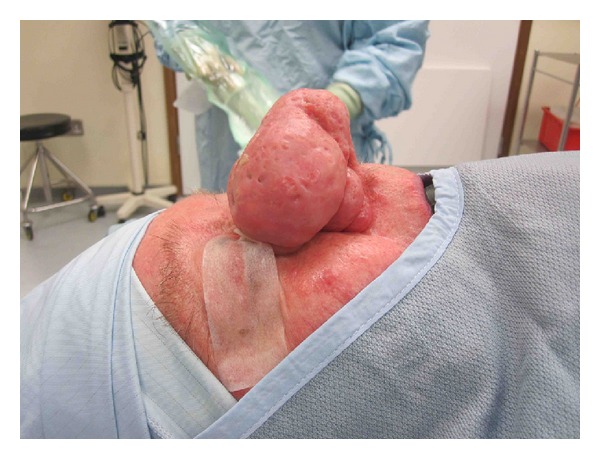
Case 3, intraoperative lateral.

**Figure 6:**
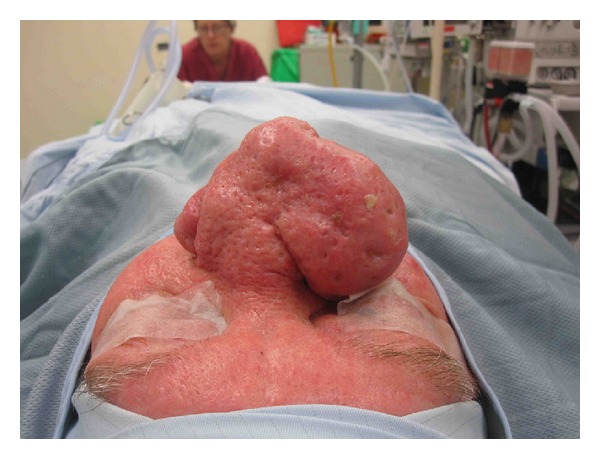
Case 3, intraoperative sky view.

**Figure 7:**
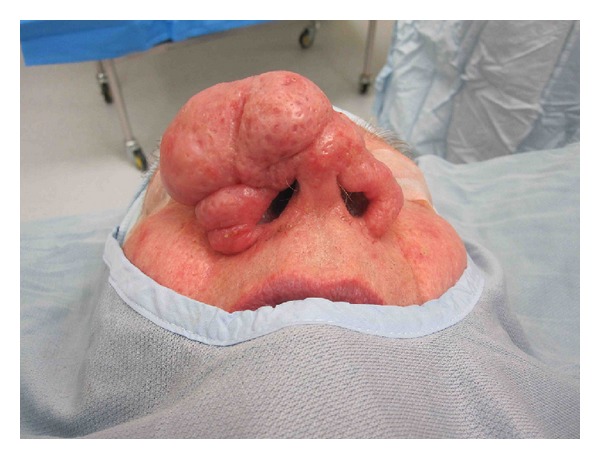
Case 3, intraoperative basal.

**Figure 8:**
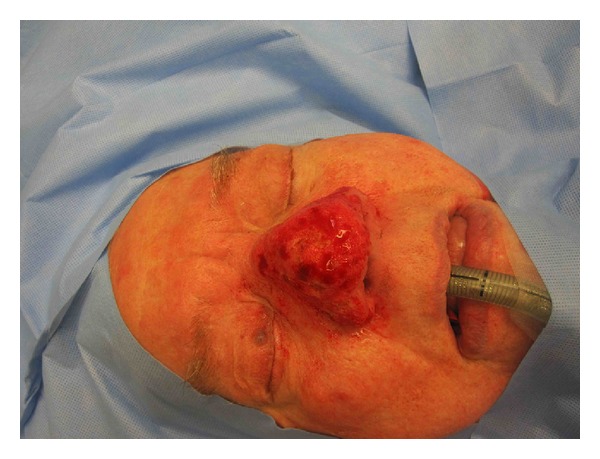
Case 3, intraoperative frontal post reduction.

**Figure 9:**
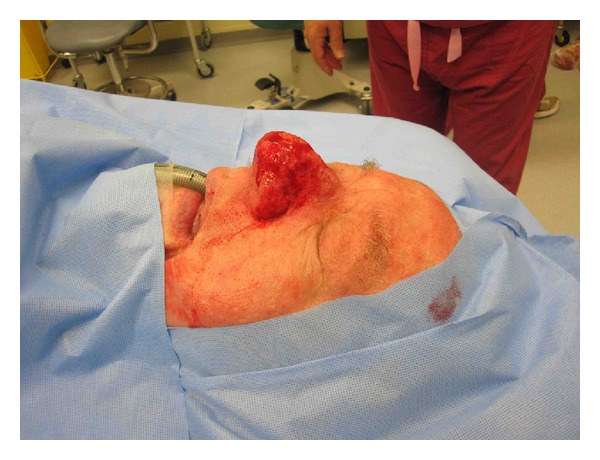
Case 3, intraoperative oblique post reduction Photos.

**Figure 10:**
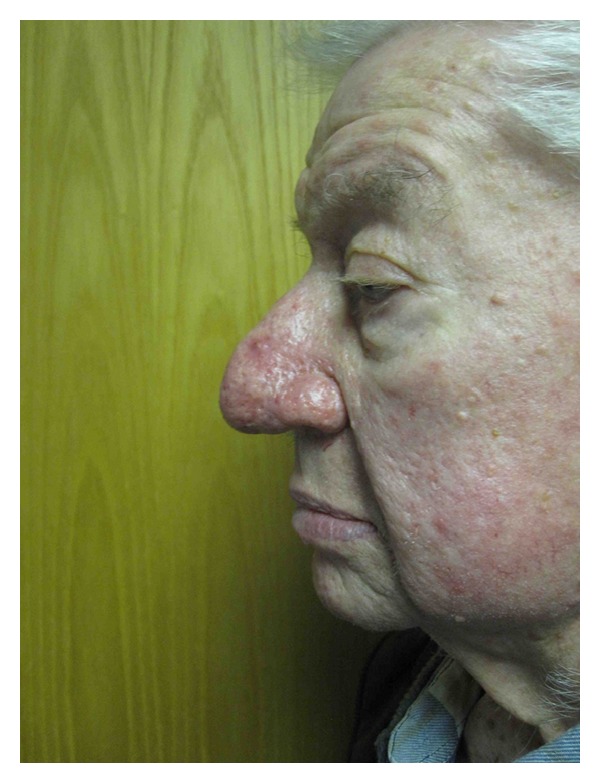
Case 3, three week postoperative lateral.

**Figure 11:**
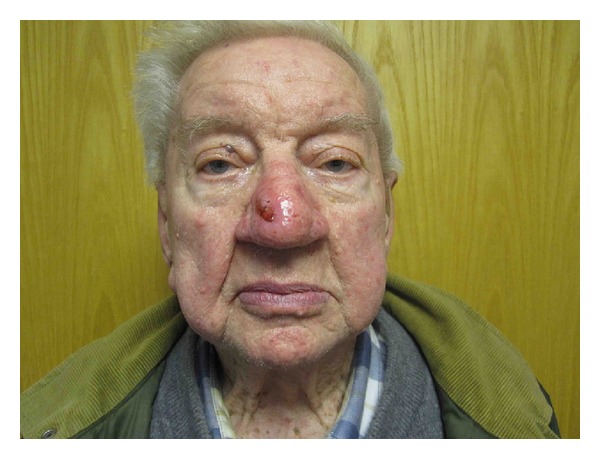
Case 3, three week postoperative frontal.

**Figure 12:**
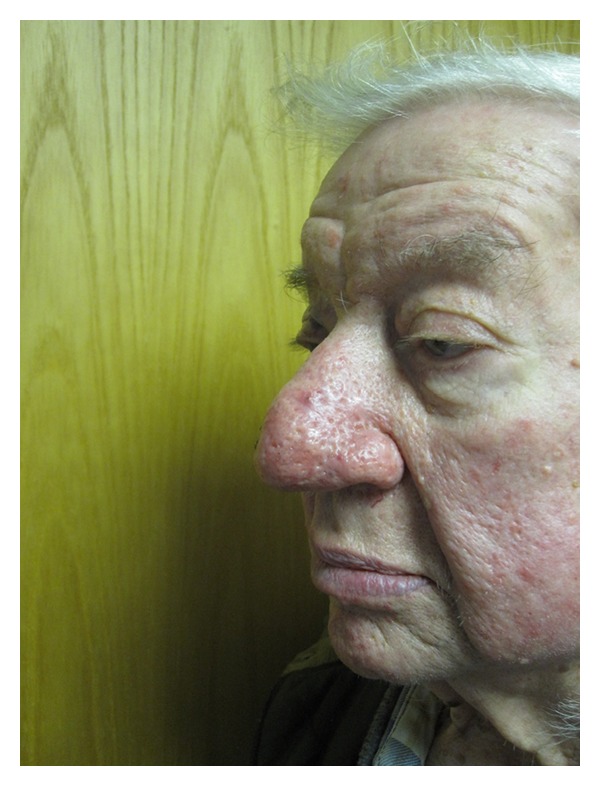
Case 3, three week postoperative oblique.

All cases were performed under general anaesthesia. The surgical field was prepared with *Betadine* an aqueous solution of 10% povidone-iodine. The nose is injected with local anesthetic lignocaine 2% with 1 in 80,000 adrenaline. Initially a ring block around the nose was performed, followed by direct infiltration into the operative field. The nose was then either initially debulked with a scalpel and then sculpted with the microdebrider (Case  3, Figures 1–12) or directly sculpted (Cases  1 and 2) with the 4 mm M4 Rotatable cutting Straight Sinus Blade on a Straightshot M4 Microdebrider by Medtronic. The technique we have utilized is to stabilise the nose with one hand while the other hand grips the distal end of the 4 mm M4 Rotatable cutting Straight Sinus Blade to improve accuracy of sculpting (see Figure 1). The blade very rapidly and precisely reduces the soft tissue in a controlled fashion. In our experience we find it helpful to keep the blade flat and flush with the nose when reducing the flat subunits of the nose such as the sidewall and dorsum and move it in a fan shaped fashion. When addressing the convex subunits such as the alar and tip, the microdebrider is rotated as it travels over the convexity to again keep the blade flat and flush with the skin. Where there are concavities such as in the transitions of the subunits, from alar to side wall (supra-alar groove), the microdebrider is held in position and rotated about its long axis to recreate a smooth curved transition. The microdebrider by Medtronic has a suction irrigation through the lumen of the blade and therefore helps keep the operative field clear to allow precise sculpting and minimizes aerosol of blood. The rpm setting can be varied to allow increase or decrease in the rate of tissue debridement as in FESS. We start with at 800 rpm on a oscillate setting and vary as the case requires. In lower settings a slight increase in tissue debridement is observed <1000 rpm compared to rpm >2000 where there is a reduction in rate of debridement [3]. As with other techniques for treatment of rhinophyma, one must be vigilant and should stay above the lowest part of the pilosebaceous unit to avoid excessive removal of tissue resulting in scar formation. 

Following satisfactory sculpting haemostasis was achieved using bipolar electrocoagulation to bleeding areas at a setting of 5–10 watts. Generous amounts of chloramphenicol 1% w/w antibiotic eye ointment were then placed over raw areas, and nonadherent dressing was applied to the treated surface and secured with Elastoplast. The whole procedure required 4 minutes and 36 minutes in the two mild-moderate rhinophyma cases (Cases 1 and 2) and 66 minutes in Case 3. Dressings were removed after 48 hours, and the patients were instructed to apply daily chloramphenicol 1% w/w antibiotic eye ointment until reviewed in clinic three to five weeks postoperatively. 

## 3. Discussion

Rosacea is a common condition with classic symptoms such as intermittent or persistent patchy flushing and redness (erythema) and inflammation of the central face. It is widely believed that rhinophyma represents the last stage of certain subtypes of rosacea. However one must also be mindful of the fact that rhinophyma may also occur in patients with few or no other features of rosacea. There are four sub-types of rosacea which have differing risks of developing phymatous lesions. Erythematotelangiectatic rosacea (subtype 1), described by persistent erythema of the central face, rhinophyma occasionally coexist. Papulopustular rosacea (subtype 2) persistent erythema of the central face; and dome-shaped erythematous papules; phymatous skin changes may be present. phymatous (subtype 3) thickened skin with prominent pores, which may affect the nose (rhinophyma—most common type), chin (gnathophyma), forehead (metophyma), ears (otophyma), and eyelids (blepharophyma). Ocular rosacea (subtype 4), sensation of foreign body in the eye; telangiectasia and erythema of lid margins [2].

Phymatous tissue and therefore the occurrence of rhinophyma when present in the nose are said to occur when dilation of the follicular orifices, hypervascularity, hypertrophy of connective tissue, dermal fibrosis, and sebaceous gland hyperplasia are observed. Four variants of rhinophyma can be recognized: glandular, fibrous, fibroangiomatous, and actinic. In glandular form, the nose is enlarged mainly because of sebaceous gland hyperplasia. In the fibrous form diffuse hyperplasia of the connective exists. In the fibroangiomatous form fibrosis, telangiectasias, and inflammatory lesions are present. In the actinic form, nodular masses of elastic tissue distort the nose [4]. Although different histological subtypes do seem to occur, the diagnosis of rhinophyma is clinical. A biopsy can be useful where there is concern to rule out possible differentials such as lupus pernio, basal cell, squamous cell, sebaceous carcinomas, or nasal lymphoma [5].

A grading scheme has been suggested for rhinophyma:

1. slight puffiness of nose;
2. bulbous nasal swelling;
3. marked nasal swelling; distortion of nasal contour [2].

The treatment of rosacea is medical with topical treatment alone being usually effective for mild-to-moderate papulopustular rosacea. Topical metronidazole in combination with 10 percent sodium sulfacetamide is a therapeutic example. For patients with moderate-to-severe papulopustular rosacea (grade 2 to 3), oral medication such as a 6–12-week course of erythromycin or doxycycline is recommended [2]. Thankfully only a small number of patients with rosacea go on to progress to develop phymatous skin changes. In one study of 108 patients with rosacea only 14% of patients were suffering from rhinophyma and 93% were men [1]. Unlike in Rosacea, where medical therapy for the treatment of rosacea is often useful, no antibiotic or retinoid has been shown conclusively to halt the progression from rosacea to rhinophyma or cause regression of existing rhinophyma [6]. Therefore, the recommendation of treatment for patients with progressive rhinophyma (stage 2 or 3) is surgical intervention to prevent or minimize the functional and psychosocial issues from progressive deformity. There are however no controlled trials comparing different surgical therapies [2, 6]. There are a myriad of surgical treatments such as cold steel scalpel, razor shave, CO_2_ laser, dermabrasion, and Bovie electrocautery [7–10]. Each of these techniques has their own unique mix of advantages and disadvantages. In our practice we have used cold steel and CO_2_ laser reduction and Bovie needle electrocautery but have now converted to using microdebrider for the advantageous combination of ease of use, precision reduction of tissues and speed of surgery. 

In our experience with cold steel reduction it is difficult to precisely reproduce the gently flowing convex and concave contours of the nose with an 11 or 15 scalpel blade. This technique tends also to lead to bleeding onto the surgical field, which needs to be controlled with packs by an assistant and liberal use of intra operative bipolar diathermy and local anesthetic with adrenaline infiltration in order to obtain a clear operative field to precisely sculpt the nose. 

Electrocautery excision has greatly improved haemostasis over cold steel techniques; however, it is technically demanding to accurately sculpt the nasal tissues. The precise but rapid reduction of the nasal tissues in sequential 1-2 mm thick layers of excised nasal tissue necessary to effectively sculpt the nose is difficult to achieve with Bovie needle electrocautery and to a lesser extent cold steel techniques. It is also our experience that cauterisation effect of the cut tissue makes precise estimation of the depth of the cut in relation to the cartilage difficult, and structures such as the thin lower lateral cartilages are easily violated by the unwary. Aside from over resection, there are also theoretical concerns of inadvertent thermal damage to the remaining epithelial cells in the pilosebaceous units that are then required to repopulate the skin during the healing phase. This would possibly risk more scarring and a less aesthetic outcome.

The CO_2_ laser combines the advantages of haemostasis and the gradual precise reduction of the nasal tissues layer by layer. Unfortunately this technique does greatly prolong operative time, through laser safety set up and also by the fact that only a thin layer of nasal skin/tissue is vapourised with each pass of the CO_2_ laser. For instance, using the CO_2_ laser for Case 3 would most certainly have resulted in excessively long operative times in our hands. 

Dermabrasion allows precise rapid reduction of the nasal tissues. Our operative times ranged from 4 minutes in the mild-moderate case to 66 minutes in the severe case; however, this technique does also produces considerable intra operative bleeding. This bleeding when combined with a rotating burr or fraise produces an aerosol of blood droplets, which exposes the operating room staff to risk of transmission of blood-borne pathogens. This aerosol (and subsequent infection risk) makes this technique undesirable given the effective alternative techniques available.

 In our experience the microdebrider offers a favourable combination of advantages over other techniques. It allows rapid precise contouring of the nose. The microdebrider has a suction irrigation channel through the lumen of the blade and therefore helps keep the operative field clear, minimizes aerosol of blood, and hence assist precise sculpting. Using the oscillating setting the suction channel is less likely to become clogged with tissue, and the rpm can be varied to adjust the rate of tissue debridement. Low oscillating speeds increase the rate of debridement, and higher speeds reduce debridement rate and increase accuracy [3]. The bimanual approaches give constant tactile feedback regarding the thickness of nasal tissues. It is these combinations of factors that allow the surgeon to precisely but rapidly reduce the tissues whilst avoiding overreduction of the pilosebaceous units or violation of the nasal cartilages. Unlike in cold steel or Bovie cautery methods, where a straight blade/cutting instrument is used, the microdebrider has a curved end. This can be utilized to great effect to help sculpting of the concavities of the nose, such as in the transitions of subunits from alar to side wall (supra-alar groove), alar to cheek (alar-facial sulcus), and nares. In these areas, the microdebrider is simply held in position and rotated about its long axis to reproduce a smooth concave curve/transition. The microdebrider is straightforward to use it requires no additional training enabling the surgeon to rapidly and accurately sculpt the nose to an acceptable aesthetic result. It is already stocked in most ENT theatres and therefore can avoid the cost outlay for additional equipment. In our experience it is more precise than cold steel or Bovie cautery, quicker than CO_2_ laser techniques, and avoids the aerosol and infection risk of dermabrasion. No complications occurred in our series, and all patients rated their cosmetic outcome as excellent, and Cases 2 and 3 had complete resolution of their functional nasal obstruction.

## 4. Conclusion

This case series describes our experience with the microdebrider as a tool for correction of rhinophyma. We have found it to be is a useful tool with distinct advantages over other commonly described methods for rapid, precise, and aesthetic reduction of rhinophyma.
